# Supplementary material for: Impact of pH and High-Pressure Pasteurization on the Germination and Development of Clostridium perfringens Spores under Hyperbaric Storage versus Refrigeration
Source: Foods. 2024 Jun 11;13(12):1832. doi: 10.3390/foods13121832 (PMC11202566; doi:10.3390/foods13121832)
Supplement: Supplementary file 1 [file foods-13-01832-s001.zip › foods-3018122-supplementary/Supplementary figures.pdf]

Supplementary Materials

BHI-broth, pH 4.50, 6.00 and 7.50, and coconut water, pH 5.40

First order kinetics model:

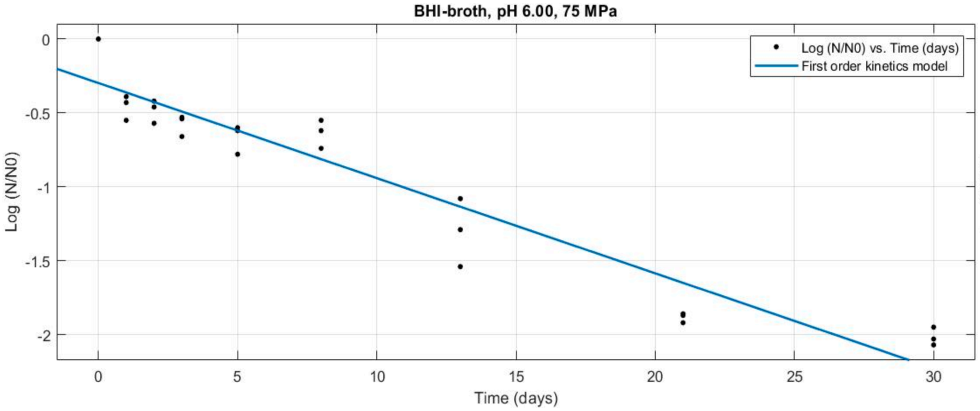

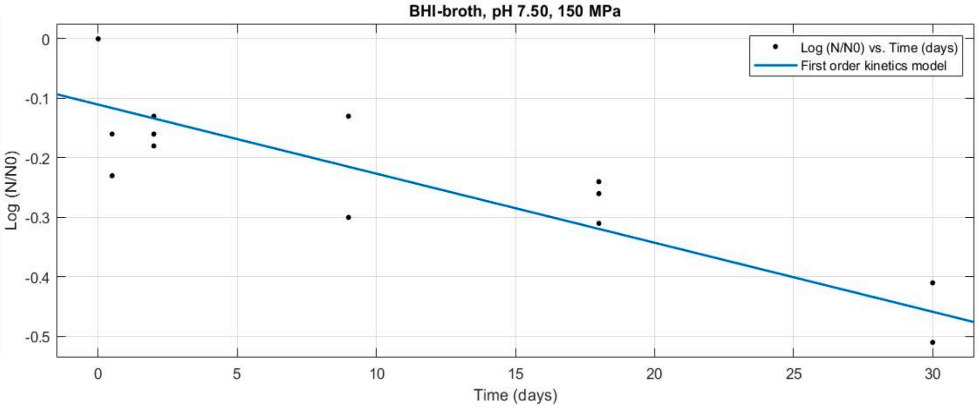

Figure S1: First order model fitting for *Clostridium perfringens* endospores inactivation by hyperbaric storage at uncontrolled room temperature (75-200 MPa, 18-23 ºC) in BHI-broth at pH 6.00 and 7.50. The red crosses indicate that the experimental data at the indicated storage times (in days) was not used for the model fitting.

Weibull model:

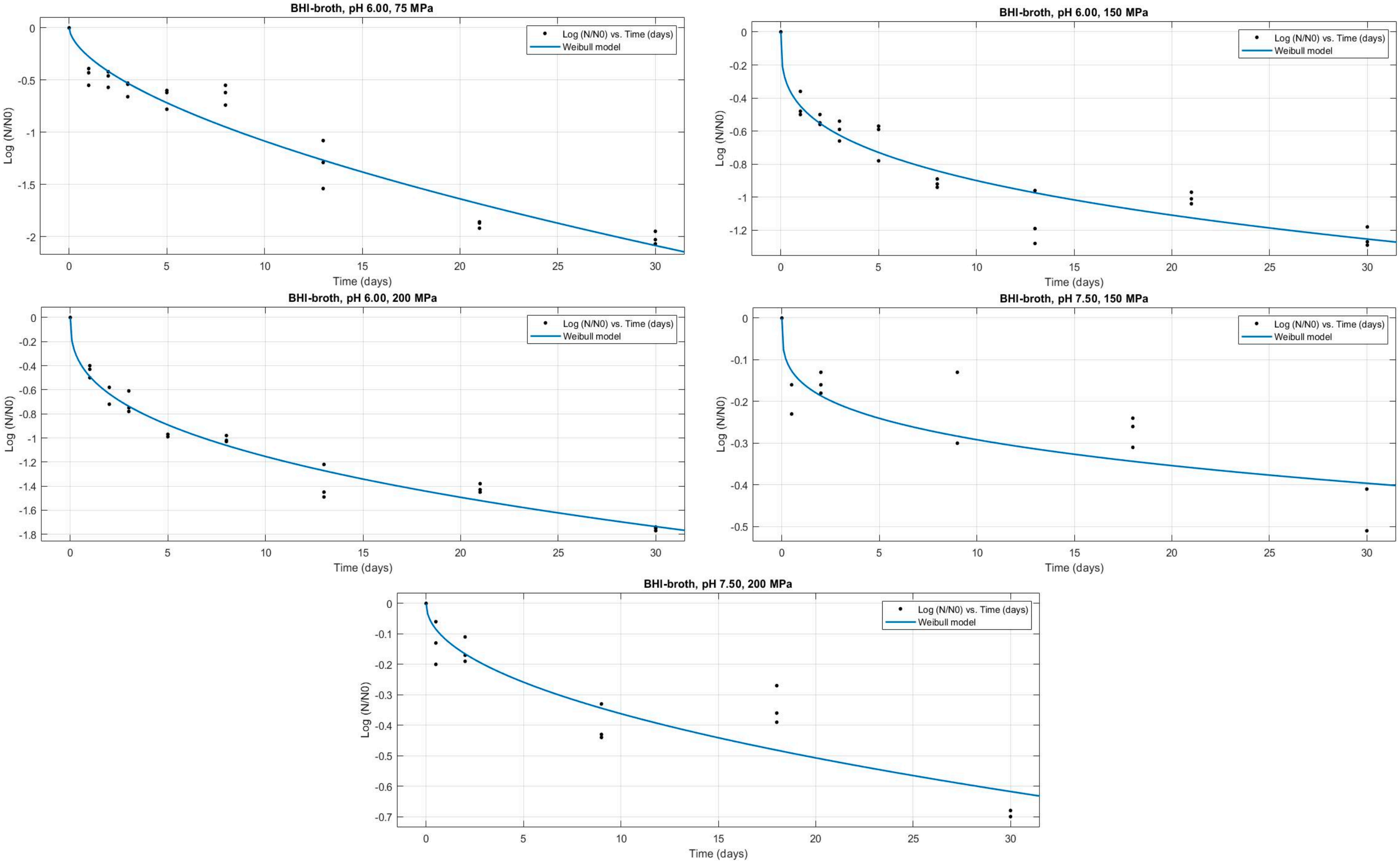

Figure S2: Weibull fitting for *Clostridium perfringens* endospores inactivation by hyperbaric storage at uncontrolled room temperature (75-200 MPa, 18-23 ºC) in BHI-broth at pH 6.00 and 7.50.

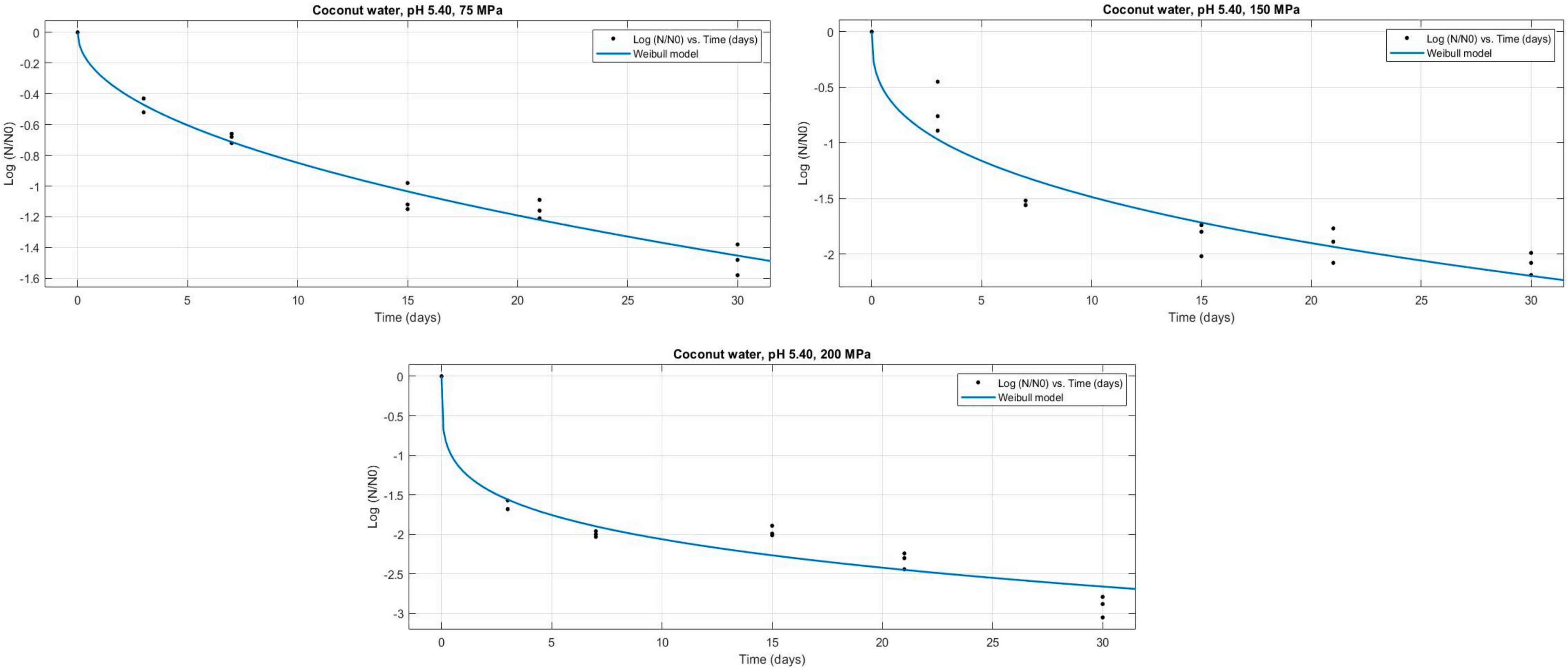

Figure S3: Weibull fitting for *Clostridium perfringens* endospores inactivation by hyperbaric storage at uncontrolled room temperature (75-200 MPa, 18-23 ºC) in coconut water, pH 5.40.

High pressure processed BHI-broth, pH 4.50, 6.00 and 7.50, and coconut water, pH 5.40

First order kinetics model:

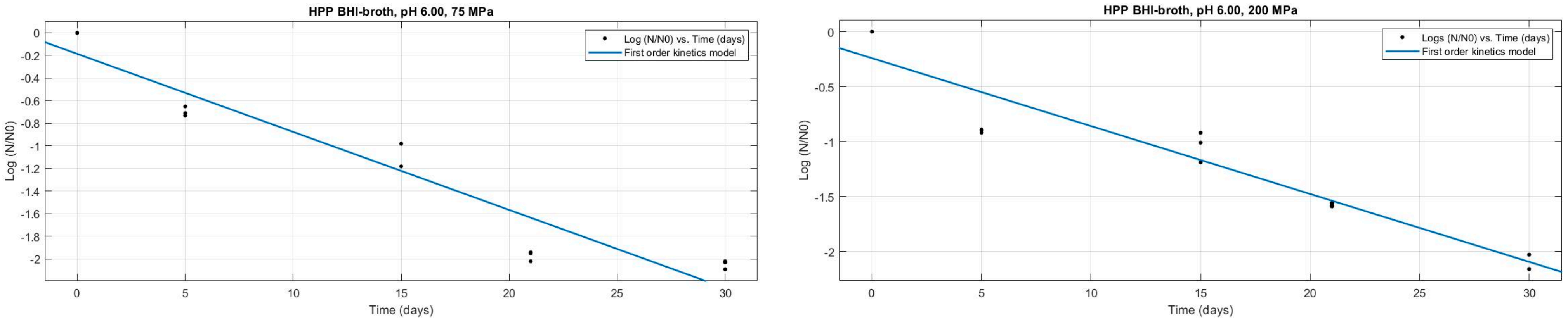

Figure S4: First order kinetics model fitting for *Clostridium perfringens* endospores inactivation by hyperbaric storage at uncontrolled room temperature (75-200 MPa, 18-23 ºC) in BHI-broth at pH 7.50 previously pasteurized by high pressure processing (600 MPa, 3 min, 17 ºC, HPP).

Weibull model:

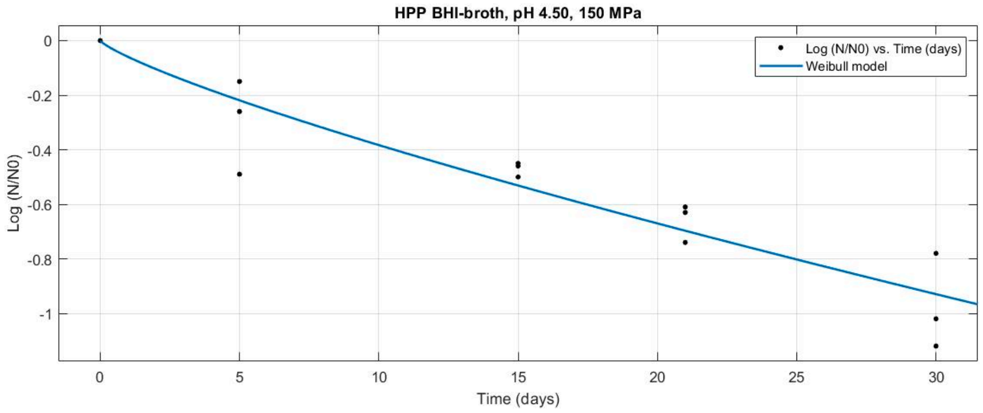

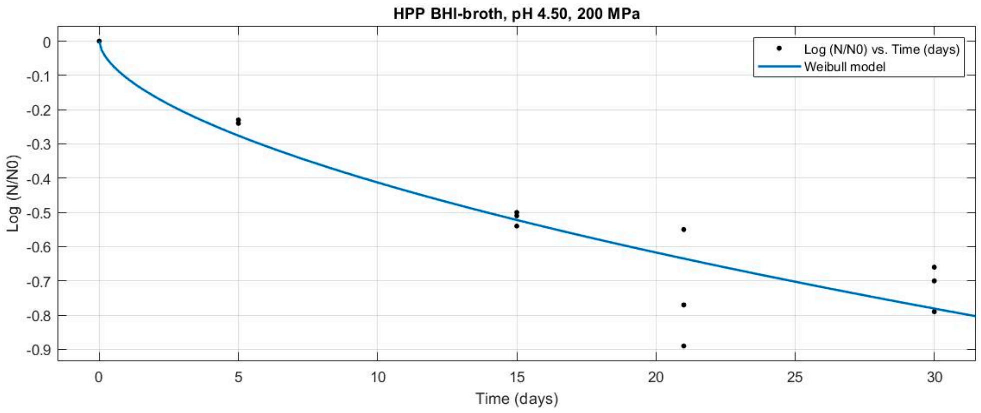

Figure S5: Weibull model fitting for *Clostridium perfringens* endospores inactivation by hyperbaric storage at uncontrolled room temperature (150 and 200 MPa, 18-23 ºC) in BHI-broth at pH 4.50 previously pasteurized by high pressure processing (600 MPa, 3 min, 17 ºC, HPP).

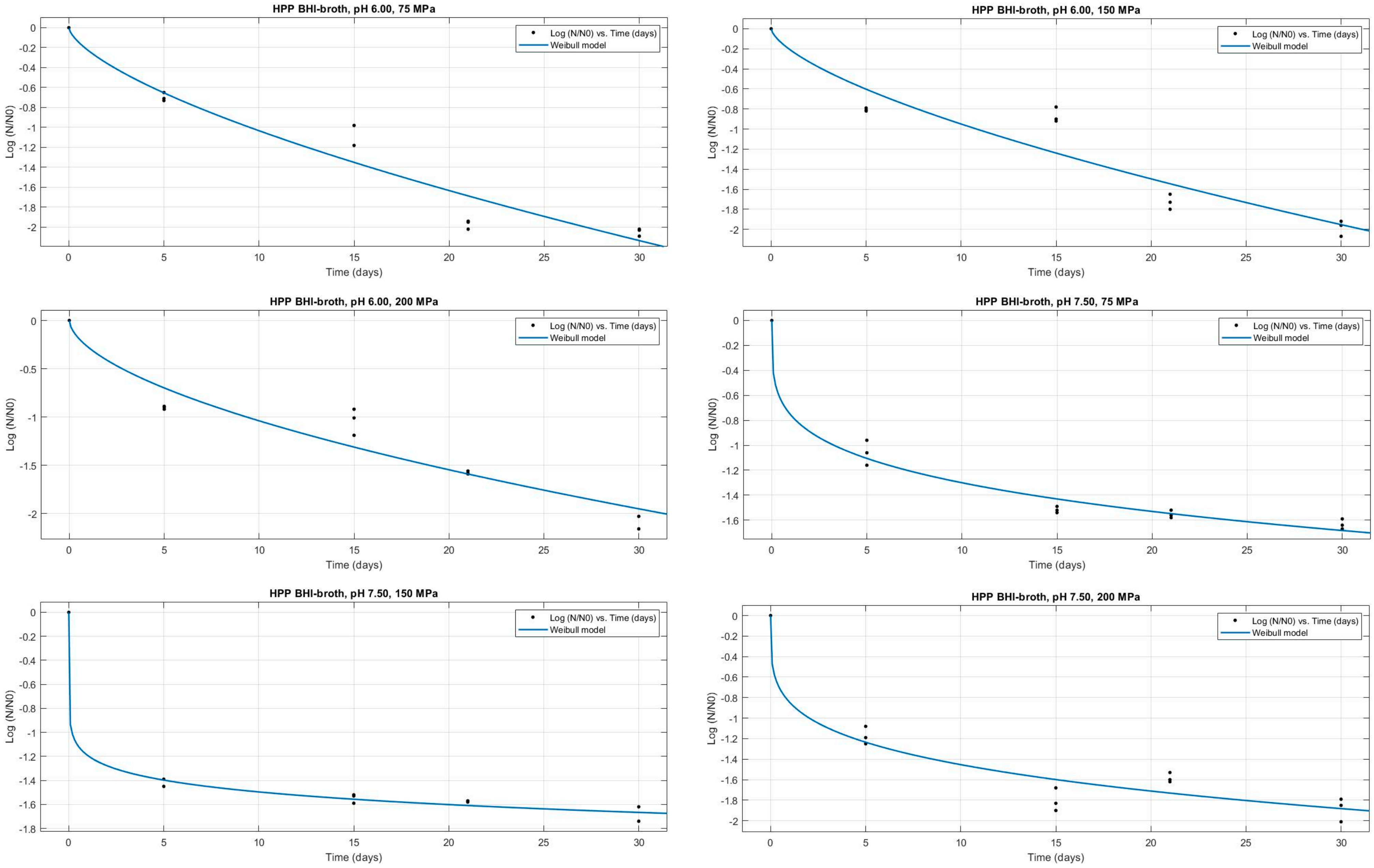

Figure S6: Weibull model fitting for *Clostridium perfringens* endospores inactivation by hyperbaric storage at uncontrolled room temperature (75-200 MPa, 18-23 ºC) in BHI-broth at pH 6.00 and 7.50 previously pasteurized by high pressure processing (600 MPa, 3 min, 17 ºC, HPP).

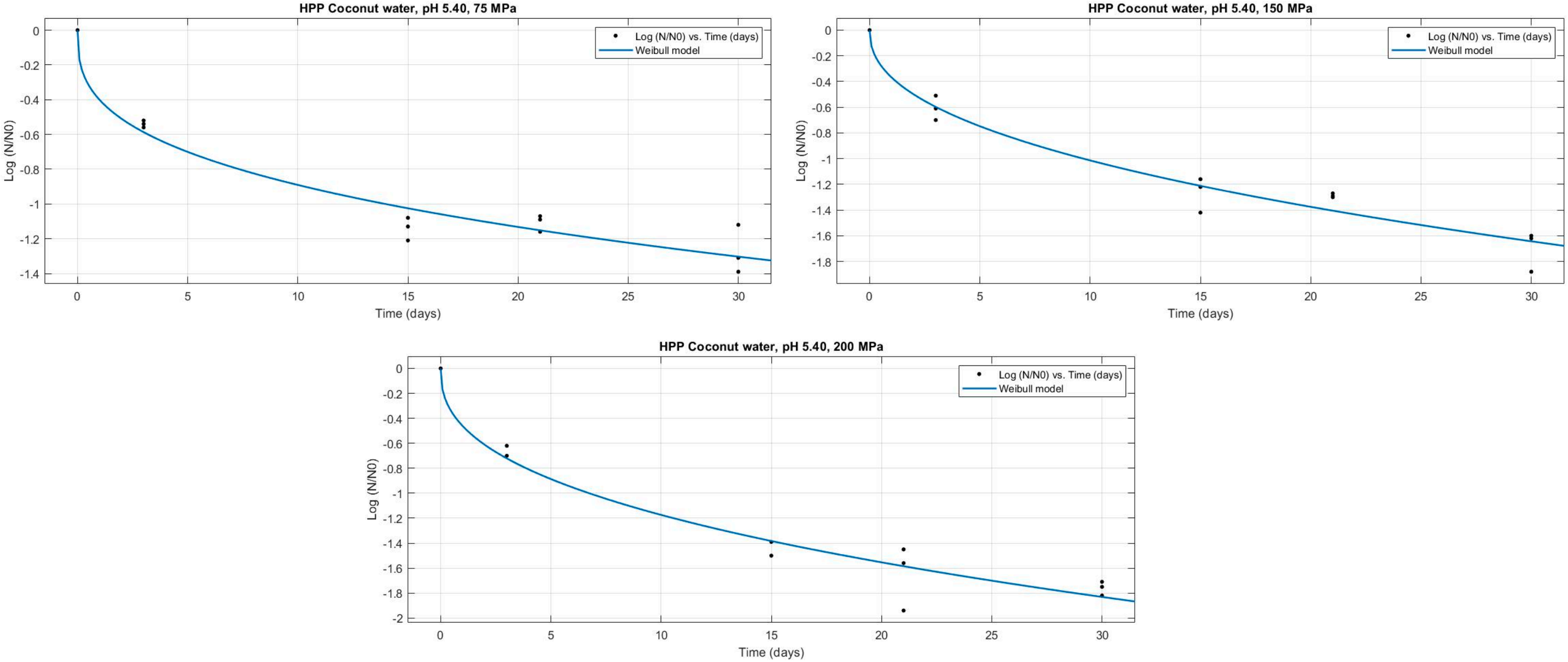

Figure S7: Weibull model fitting for *Clostridium perfringens* endospores inactivation by hyperbaric storage at uncontrolled room temperature (75-200 MPa, 18-23 ºC) in coconut water, pH 5.40, previously pasteurized by high pressure processing (600 MPa, 3 min, 17 ºC, HPP).

BHI-broth, pH 4.50, 6.00 and 7.50, No activation

First order kinetics model

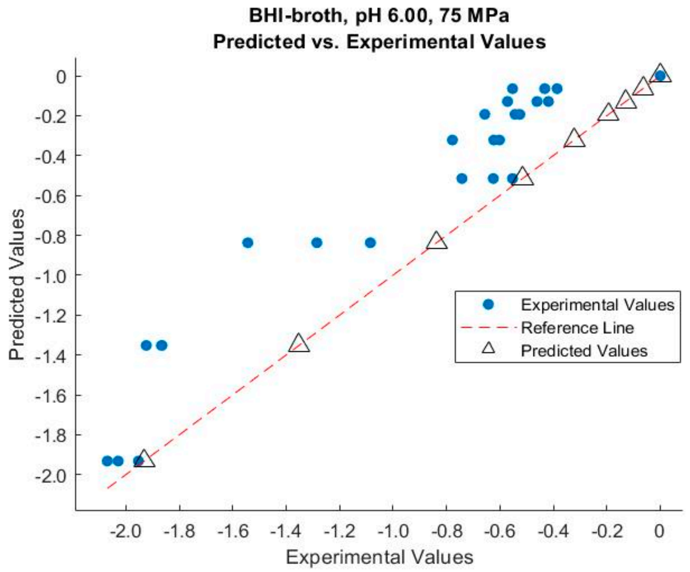

Figure S8: Experimental versus predicted values derived from the first order kinetics model describing the inactivation of *Clostridium perfringens* endospores by hyperbaric storage at uncontrolled room temperature (75-200 MPa, 18-23 ºC) in brain-heart infusion broth (pH 4.50 and 6.00, BHI-broth). The blue circles indicate the experimental values, while the triangles indicate the values predicted by the model.

Weibull model:

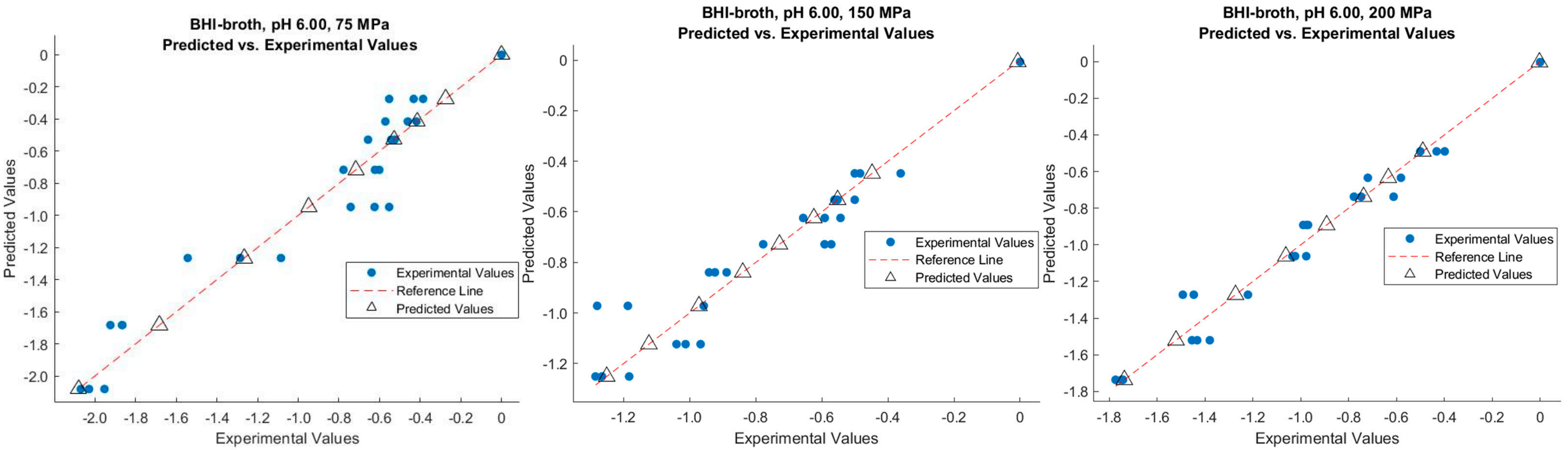

Figure S9: Experimental versus predicted values derived from the Weibull model describing the inactivation of *Clostridium perfringens* endospores by hyperbaric storage at uncontrolled room temperature (75-200 MPa, 18-23 ºC) in brain-heart infusion broth (pH 4.50 and 6.00, BHI-broth). The blue circles indicate the experimental values, while the triangles indicate the values predicted by the model.

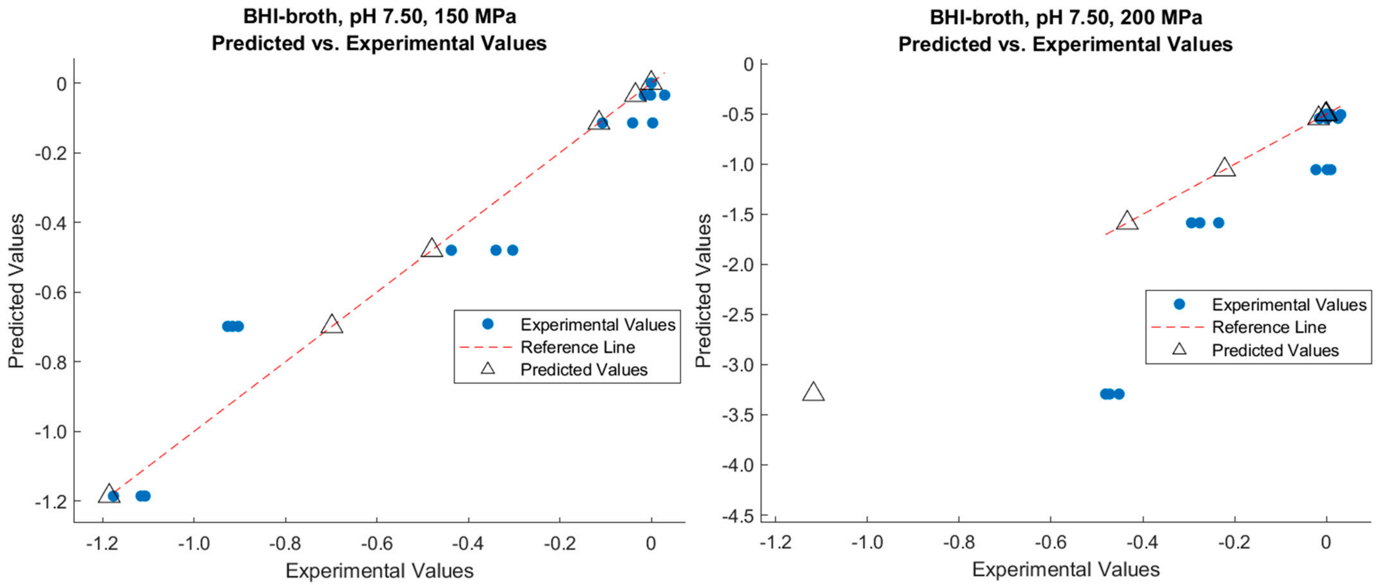

Figure S10: Experimental versus predicted values derived from the Weibull model describing the inactivation of *Clostridium perfringens* endospores by hyperbaric storage at uncontrolled room temperature (75-200 MPa, 18-23 ºC) in brain-heart infusion broth (pH 7.50, BHI-broth). The blue circles indicate the experimental values, while the triangles indicate the values predicted by the model.

Coconut water, pH 5.40, No activation

Weibull model:

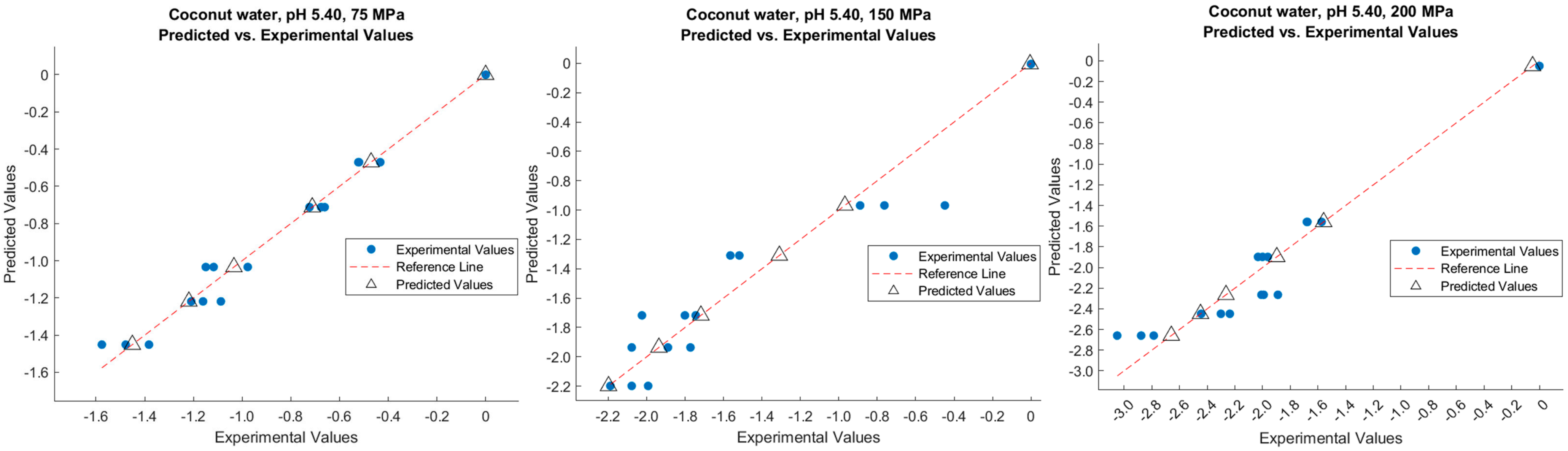

Figure S11: Experimental versus predicted values derived from the Weibull model describing the inactivation of *Clostridium perfringens* endospores by hyperbaric storage at uncontrolled room temperature (75-200 MPa, 18-23 ºC) coconut water (pH 5.40). The blue circles indicate the experimental values, while the triangles indicate the values predicted by the model.

BHI-broth, pH 4.50, 6.00 and 7.50, HPP activation

First order kinetics model:

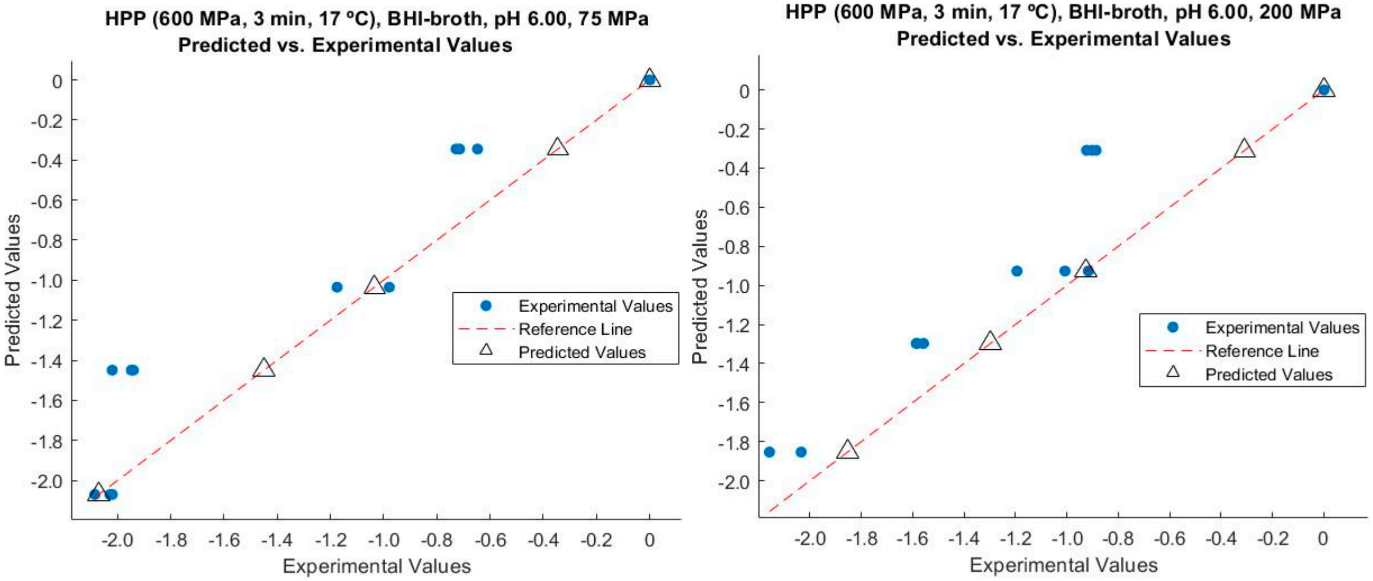

Figure S12: Experimental versus predicted values derived from the first order kinetics model describing the inactivation of *Clostridium perfringens* endospores by hyperbaric storage at uncontrolled room temperature (75-200 MPa, 18-23 ºC) in BHI-broth (pH 4.50 and 6.00) pre-activated by high pressure processing (600 MPa, 3 min, 17 ºC, HPP). The blue circles indicate the experimental values, while the triangles indicate the values predicted by the model.

Weibull model:

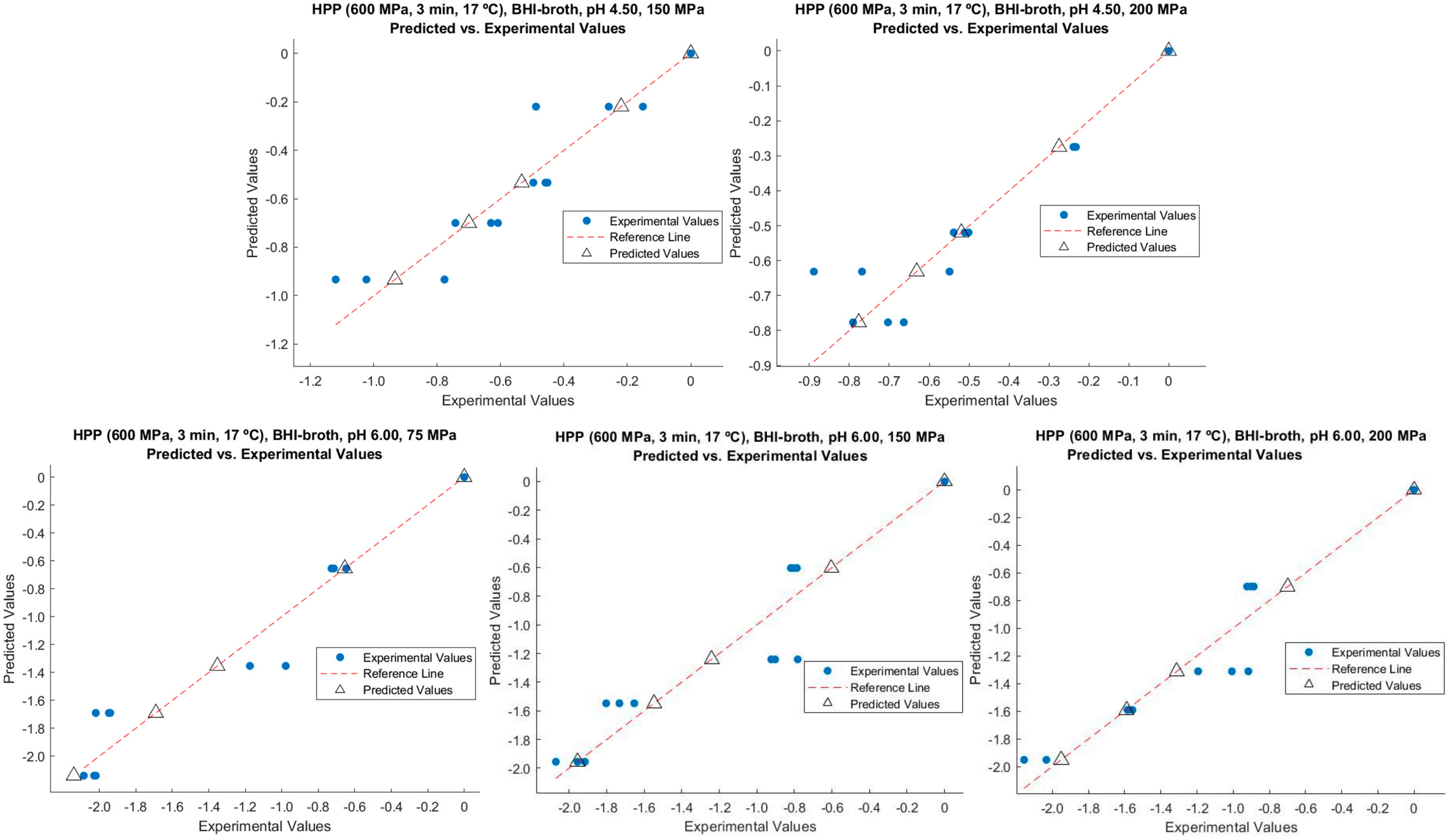

Figure S13: Experimental versus predicted values derived from the Weibull model describing the inactivation of *Clostridium perfringens* endospores by hyperbaric storage at uncontrolled room temperature (75-200 MPa, 18-23 ºC) in BHI-broth (pH 4.50 and 6.00) pre-activated by high pressure processing (600 MPa, 3 min, 17 ºC, HPP). The blue circles indicate the experimental values, while the triangles indicate the values predicted by the model.

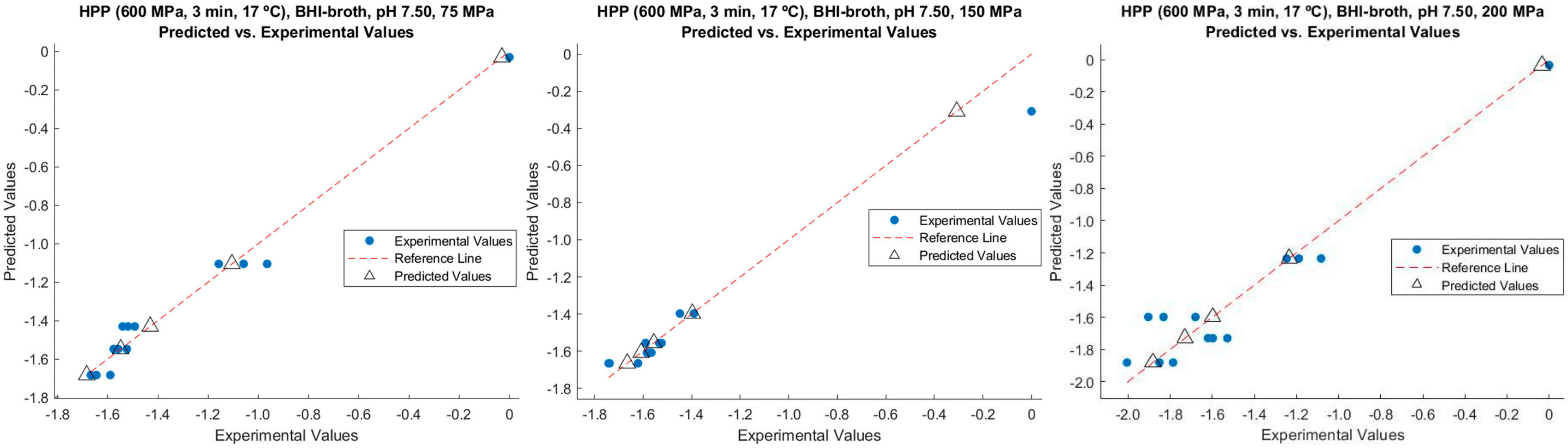

Figure S14: Experimental versus predicted values derived from the Weibull model describing the inactivation of *Clostridium perfringens* endospores by hyperbaric storage at uncontrolled room temperature (75-200 MPa, 18-23 ºC) in BHI-broth (pH 7.50) pre-activated by high pressure processing (600 MPa, 3 min, 17 ºC, HPP). The blue circles indicate the experimental values, while the triangles indicate the values predicted by the model.

Coconut water, pH 5.40, HPP activation

Weibull model:

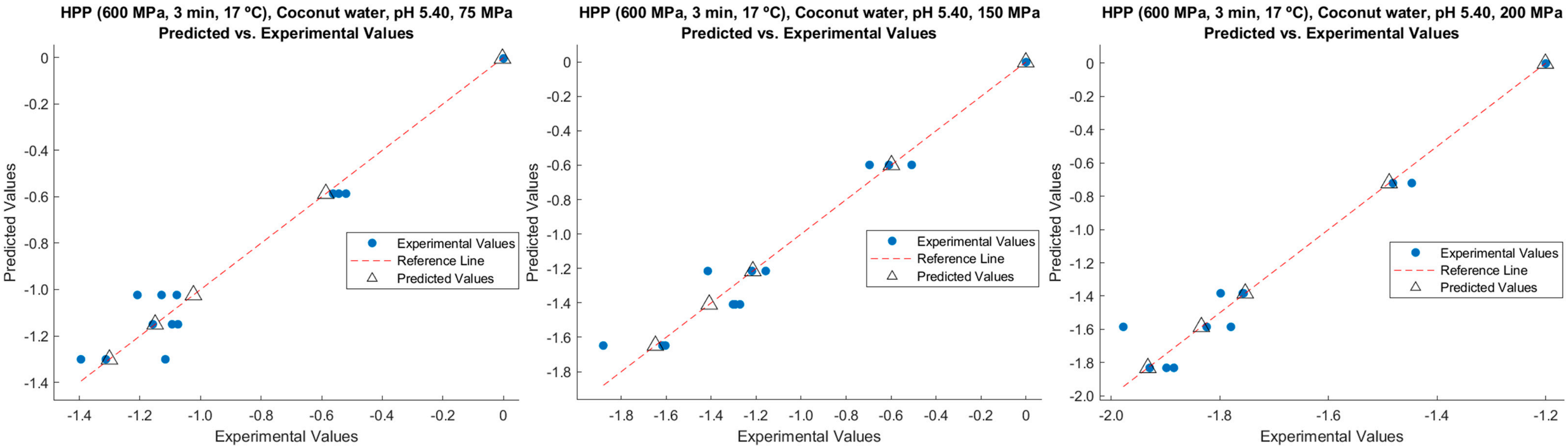

Figure S15: Experimental versus predicted values derived from the Weibull model describing the inactivation of *Clostridium perfringens* endospores by hyperbaric storage at uncontrolled room temperature (75-200 MPa, 18-23 ºC) in coconut water (pH 5.40) pre-activated by high pressure processing (600 MPa, 3 min, 17 ºC, HPP). The blue circles indicate the experimental values, while the triangles indicate the values predicted by the model.
