# Supplementary material for: Impact of pH and High-Pressure Pasteurization on the Germination and Development of Clostridium perfringens Spores under Hyperbaric Storage versus Refrigeration
Source: Foods. 2024 Jun 11;13(12):1832. doi: 10.3390/foods13121832 (PMC11202566; doi:10.3390/foods13121832)
Supplement: Supplementary file 1 [file foods-13-01832-s001.zip › foods-3018122-supplementary/Supplementary materials.pdf]

Supplementary materials

Table S1: Total microbial loads (TML) and heat resistant endospore fraction (HREF) at the beginning of each storage experiment. The values are presented as mean ± standard deviation.

Matrix	pH	Storage conditions	Initial TML (Log CFU/mL)	Initial HREF (Log CFU/mL)
**BHI-broth**	4.50	AP/RT, RF and HS (75, 150 and 200 MPa)	6.34 ± 0.10	5.64 ± 0.07
	6.00		5.58 ± 0.11	5.12 ± 0.08
	7.50		5.94 ± 0.05	5.47 ± 0.07
**HPP-BHI-broth**	4.50		5.94 ± 0.08[a]<br>5.12 ± 0.05	5.88 ± 0.06[a]<br>4.23 ± 0.06
	6.00		6.58 ± 0.11[a]<br>6.21 ± 0.13	6.38 ± 0.03[a]<br>5.41 ± 0.10
	7.50		6.43 ± 0.05[a]<br>6.15 ± 0.07	6.21 ± 0.09[a]<br>5.08 ± 0.10
**Coconut water**	5.40	AP/RT, RF and HS (75, 150 and 200 MPa)	6.10 ± 0.04	5.77 ± 0.12
**HPP-Coconut water**			5.11 ± 0.05[a]<br>4.79 ± 0.02	4.82 ± 0.07[a]<br>3.37 ± 0.04

[a]Initial load before the HPP treatment
